# Prices of medicines for the management of pain, diabetes and cardiovascular diseases in private pharmacies and the national health insurance in Tanzania

**DOI:** 10.1186/s12939-020-01319-9

**Published:** 2020-11-10

**Authors:** Rashid Bakari Kirua, Mary Justin Temu, Amani Thomas Mori

**Affiliations:** 1 Department of Pharmaceutics, School of Pharmacy, Muhimbili University of Health and Allied Sciences, P.O. Box 65013, Dar es Salaam, Tanzania; 2 Pharmct Council-Ministry of Health, Community Development, Gender, Elderly, and Children, P.O. Box 31818, Dar es Salaam, Tanzania; 3 Department of Global Public Health and Primary Care, University of Bergen, P.O. Box 7804, 5020 Bergen, Norway

**Keywords:** Essential medicines, Prices, Reference prices, Private pharmacy, Tanzania

## Abstract

**Background:**

High price is a major challenge limiting access to essential medicines especially among the poorest families in developing countries. The study aims to compare the prices of medicines used in the management of pain, diabetes, and cardiovascular diseases in private pharmacies and the National Health Insurance Fund (NHIF) in Tanzania. Pharmacy prices were also compared with the prices of medicines surveyed nationally by WHO/HAI in 2012.

**Method:**

This cross-sectional study was conducted in Dar es Salaam, Morogoro, Dodoma, and Kilimanjaro regions from February to April 2015. Data were collected from 33 private pharmacies, NHIF and, the HAI database. The study used the WHO/HAI methodology. The analysis was done using non-parametric Kruskal-Wallis and post-hoc pair-wise comparison Dunn test, while a possible change in prices between our survey and 2012 WHO/HAI national survey data was tested using a Sign test in Stata version 16.1.

**Results:**

Twenty-eight essential medicines, of which 9 are used for management of pain, 7 for diabetes, and 12 for cardiovascular diseases were analyzed. There was a significant difference in the mean pharmacy prices of some medicines between the regions and between the pharmacies and NHIF reference prices. NHIF reference prices were higher than the pharmacy prices for 16 of the 28 medicines. There was a significant increase in the prices of 5 out of the 8 medicines that were also nationally surveyed by the WHO/HAI in 2012.

**Conclusion:**

The study found that medicine prices in private pharmacies vary a lot between the study regions, which raises equity concerns. Also, there was a significant difference between the pharmacy and the NHIF reimbursement prices, which may expose patients to fraudulent co-payments or hinder timely access to prescribed medicines. Therefore, effective price control policies and regulations for medicines are warranted in Tanzania.

## Introduction

Over the past two decades, there has been a substantial epidemiological transition of diseases from communicable to non-communicable diseases (NCDs). It is estimated that NCDs kill more than 40 million people annually, which is almost two-thirds of all deaths [1]. The Global Burden of Disease study ranked diabetes, low back pain, and cardiovascular diseases such as ischemic heart diseases and stroke higher in the top 25 causes of disability-adjusted life years (DALYs) [2]. The burden of NCDs in developing countries where it is estimated that 80% of the total deaths from NCDs takes place, has been rising rapidly [3]. The reasons behind the rapid epidemiological transition in developing countries have been likened to unhealthy lifestyles such as lack of physical activities, unhealthy diets, alcohol, and tobacco use [4–6].

Unlike infectious diseases, NCDs such as diabetes and cardiovascular diseases do not disable a person instantly, but progress slowly and if not managed early and effectively can lead to even more severe disabling effects in the long-term [7]. Health expenditures also increase dramatically with the increase in the number of chronic conditions affecting the patient because of the complexity of care [8]. Thus, the long-term health costs required for the management of NCDs that often include lengthy and expensive treatments can quickly drain household resources particularly in low-income settings where out-of-pocket health payments are common [9]. Governments in low- and middle-income countries are increasingly implementing alternative financing strategies including health insurance in an attempt to improve access to healthcare and to protect families from impoverishing health expenditures [10–12].

In December 2012, the United Nations General Assembly adopted a resolution on universal health coverage, which among other things urged countries to provide affordable and quality healthcare to all [13]. Today, access to essential medicines remains a major challenge to a large proportion of poor sections of populations in developing countries, especially among those suffering from NCDs. This problem is exacerbated by the high prices of essential medicines, which hinder access to care [14]. Several cost-containment measures to prevent further escalation of treatment costs have been proposed by the WHO including; regulation of mark-ups in the pharmaceutical supply chain, exemptions or reduction of tax for essential medicines, use of reference pricing, promoting the use of generics, and the use of health technology assessment to inform pricing and reimbursement decisions [15]. Nevertheless, the lack of technical capacity, supporting policy frameworks, and relevant data have hampered the effective implementation of these cost-containment strategies in most developing countries [16–18].

### The situation in Tanzania

Tanzania is an East African country with more than 45 million people [19]. The health system, which is largely public-owned, is organized in a pyramidal structure in which the primary health facilities are at the base and the specialized and referral hospitals are at the apex. A comprehensive network of primary healthcare facilities i.e. dispensaries and health centers serve most of the population. Each facility offers pharmacy services, however, due to frequent stock-outs patients often buy medicines in the private pharmacies and Accredited Drug Dispensing Outlets (ADDO). The later are authorized to stock a limited list of prescription-only-medicines and are mainly located in underserved rural areas where there are no pharmacies [20].

In 1999, the Tanzanian government established the National Health Insurance Fund (NHIF), which by then it was a compulsory health insurance scheme for serving central government employees only. However, since 2002 membership has been extended to include all public servants, private sector institutions, individuals, and their legal dependents. NHIF also manages the Community Health Fund, which mainly serves the rural populations and informal sector. As of December 2019, the number of beneficiaries was about 4.9 million people, equivalent to 9% of the total population [21].

Because of this rapid increase of the beneficiaries and low availability of medicines in public health facilities, the NHIF accredits private sector health facilities including pharmacies and ADDOs to provide medicines to its members. Medicine prices in private pharmacies and drug shops are unregulated, hence prices tend to vary depending on buying price, operational cost, and profit margin desired unlike in public health facilities where prices are relatively uniform because they get most of their medical supplies from the Medical Store Department. Also, NHIF does not usually update its reimbursement rates frequently, which may take more than 3 years [22]. This means that the actual costs incurred by providers can be much higher than the reimbursement rates, creating fraudulent co-payments that may limit timely access to prescribed medicines, and further exposing patients to catastrophic out-of-pocket health expenditures. Therefore, The study aims to compare the prices of medicines used in the management of pain, diabetes, and cardiovascular diseases in private pharmacies and the National Health Insurance Fund (NHIF) in Tanzania. It further compares pharmacy prices with the prices of medicines surveyed nationally by WHO/HAI in 2012.

## Methodology

### Study design and context

**Fig. 1:**
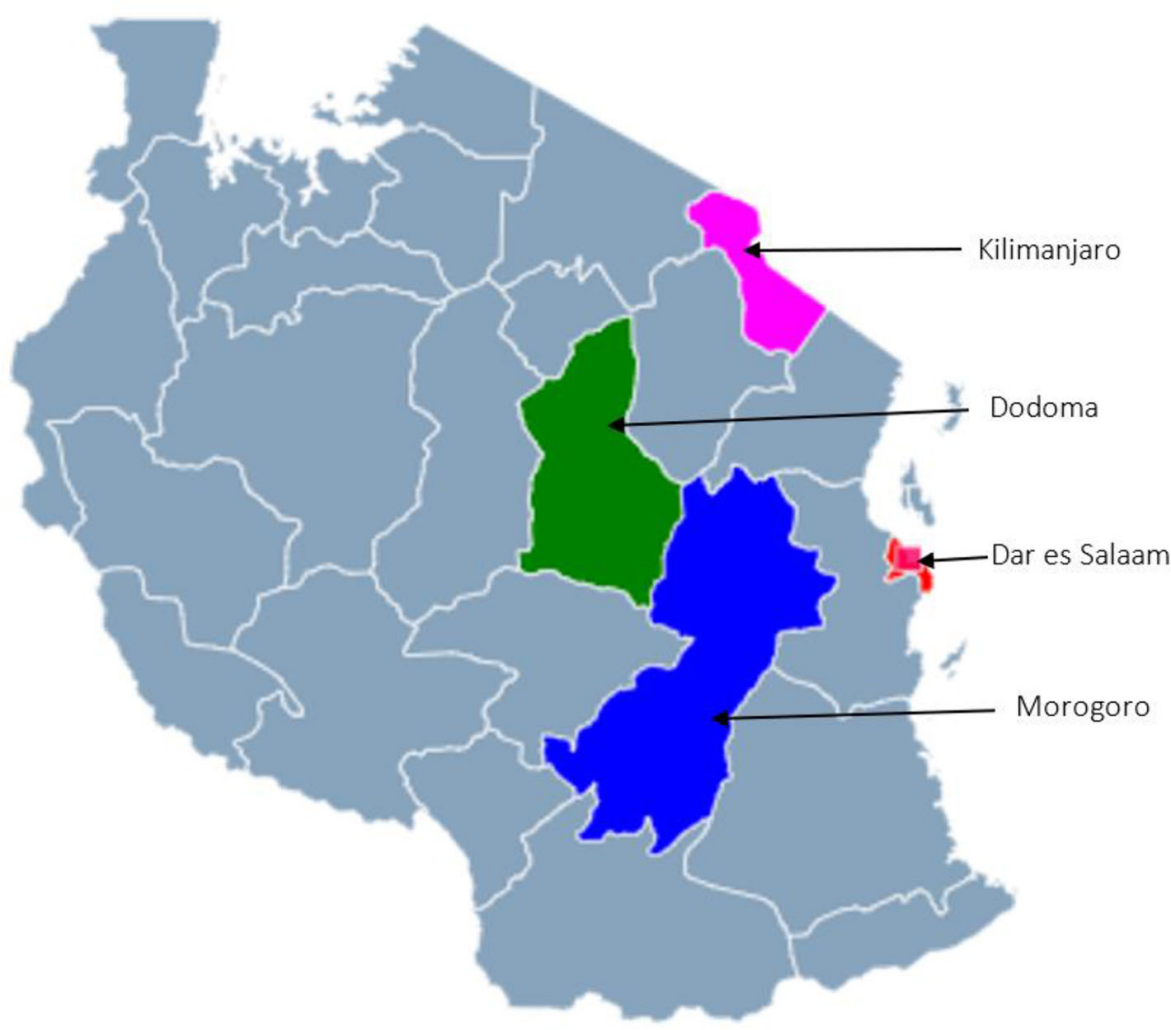
Study regions

This was a cross-sectional study, which was conducted in four regions of Tanzania, namely Dar es Salaam, which is located in the coastal zone, Morogoro and Dodoma in the central zone, and Kilimanjaro in the northern zone (Fig. 1). The study used the World Health Organization (WHO)/Health Action International (HAI) methodology designed for measuring medicine prices, availability, and affordability [23]. Data on the selected list of essential medicine was collected from 33 private pharmacies that were selected purposively from the list of the NHIF-accredited pharmacies available in the four study regions.

Dar es Salaam was selected because it is the main business city in Tanzania with a total population of about 4.3 million people (based on the 2012 national census). It is one of the smallest regions with an area of about 1393 km^2^. Among the 470 retail private pharmacies in this region, 57 pharmacies have been accredited to provide service to NHIF beneficiaries, and only 20 pharmacies were surveyed during this study. More than half of the private pharmacies in the country are in this region. Morogoro region has a population of about 2.2 million people and is located about 192 km from Dar es Salaam. Morogoro had 20 retail private pharmacies, and only 4 out of 6 accredited pharmacies were surveyed. Dodoma Region is the capital of Tanzania, with a population of about 2.1 million people. Dodoma has about 23 private pharmacies and 5 out of the 9 accredited pharmacies were surveyed. Kilimanjaro region has a population of about 1.6 million people and is about 566 km from Dar es Salaam. Kilimanjaro had about 23 private pharmacies and 4 out of 6 accredited private pharmacies were surveyed.

### Sampling of pharmacies

The purposeful sampling technique was used to select 33 private pharmacies that have been accredited to provide services to NHIF beneficiaries. Purposeful sampling seeks information-rich cases relevant to the study. The premises were selected from the three districts of Dar es Salaam region i.e. Ilala, Kinondoni, and Temeke, and from one district in each of the other three surveyed regions. Pharmacies that were located near the Regional or District Hospitals were chosen. The public hospitals usually experience frequent stock-out, hence we expected many patients would seek medicines in the nearby private pharmacies.

### Data collection and analysis

We selected 53 essential medicines that are recommended for the management of pain, diabetes, and cardiovascular diseases from the Standard Treatment Guidelines and the National Essential Medicine List (STG/NEML) [24]. We ensured that the selected essential medicines were also on the NHIF reimbursement list. Data were collected on the same dosage form and strength for each medicine. Data collection tools were piloted at Kibaha District Council in the Coast region, which was chosen because of its proximity to Dar es Salaam and the presence of Tumbi regional hospital that is surrounded by NHIF-accredited private pharmacies.

Data collection was conducted between February and April 2015 and were analyzed in the Excel spreadsheet (Microsoft Excel®, Microsoft Corporation) and Stata version 16.1. Price data were not normally distributed, hence we conducted the non-parametric Kruskal-Wallis and Dunn’s pairwise-comparison test with Bonferroni adjustment [25], to establish whether pharmacy price for each medicine was statistically different between the regions and NHIF. The Sign test was used to compare the median pharmacy prices from our survey and the WHO/HAI prices for the 2012 survey, to establish if prices have changed over the 3 years. Only the tracer medicines that were available in more than ten pharmacies were included in the analysis.

## Results

### Medicines for management of pain (antipyretics)

**Fig. 2:**
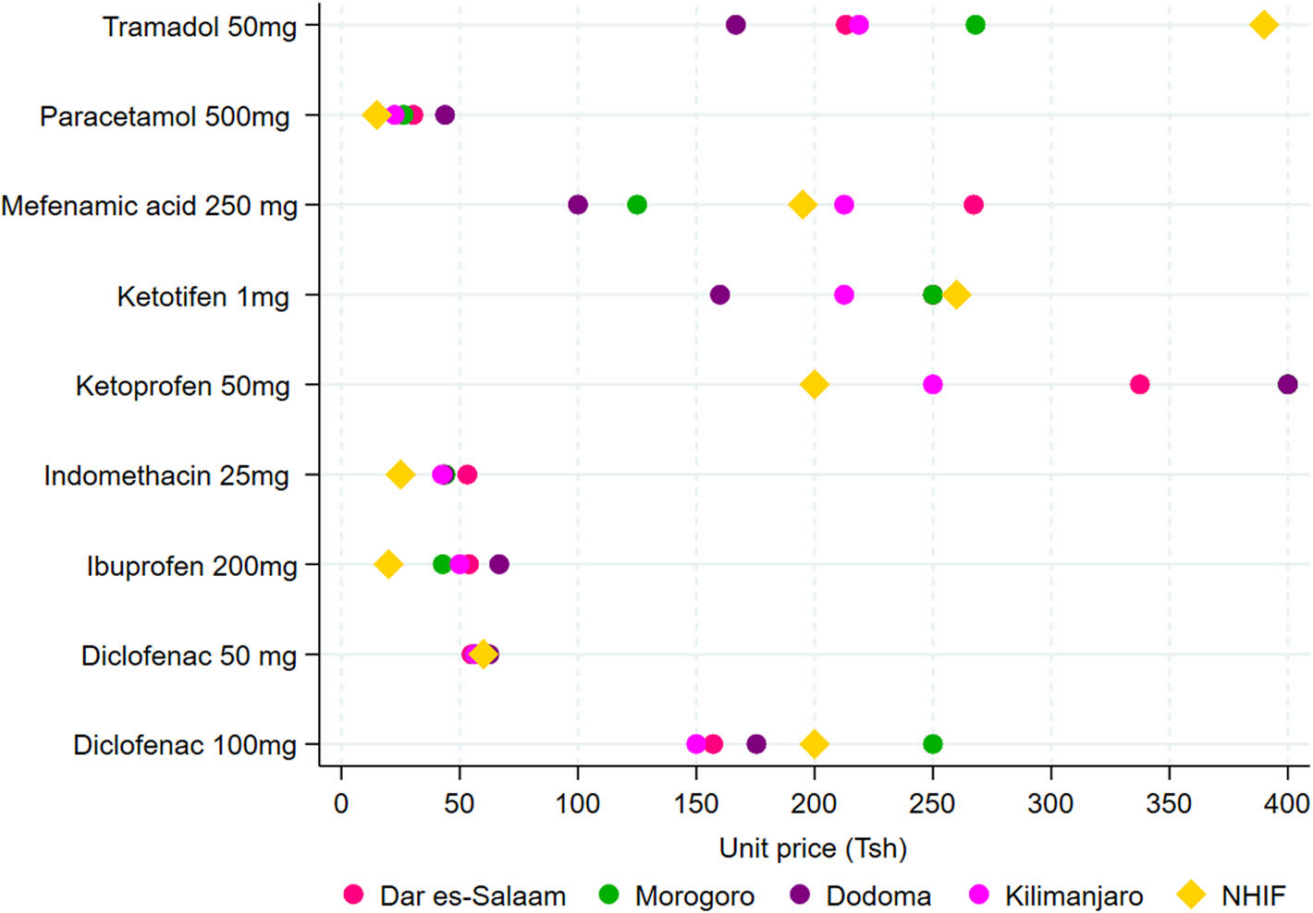
Pharmacy and NHIF reference prices for antipyretic medicines

Figure 2 shows the mean pharmacy prices of 9 antipyretic medicines in the four regions and the NHIF reference price. Prices for relatively cheap antipyretic medicines such as diclofenac 50 mg, ibuprofen 200 mg, and indomethacin 25 mg, were not significantly different in between the regions compared to relatively more expensive antipyretic medicines. However, a pair-wise post-hoc Dunn test with Bonferroni adjustment indicated a significant (*p* < 0.05) price difference for diclofenac 100 mg between Morogoro (250 Tsh), Dar es-Salaam (157.1 Tsh), and Kilimanjaro (150 Tsh); for mefenamic acid 250 mg, a large difference was observed between Dar es -Salaam (267.2 Tsh), Morogoro (125 Tsh) and Dodoma (100 Tsh).

When compared to NHIF prices, a pair-wise post-hoc Dunn test with Bonferroni adjustment indicated a significant (*p* < 0.05) price difference between NHIF and pharmacy price for all medicines. NHIF price for ibuprofen tablets 200 mg was lower than pharmacy price in all regions, while for tramadol 250 mg it was higher than in all regions except Morogoro. For indomethacin capsules 25 mg the NHIF price was lower than pharmacy prices in Dar es Salaam and Morogoro, while the price for paracetamol tablets 500 mg was significantly higher than the NHIF reference price in Dar es Salaam by 15.4 Tshs and Dodoma by 28.8 Tsh. NHIF price for ketoprofen 50 mg was significantly lower than the pharmacy prices in Dar es Salaam (337.5 Tshs) and Dodoma (400 Tsh) and Morogoro (400 Tsh) regions. (see Additional file 1).

### Medicines used in the management of cardiovascular diseases

**Fig. 3:**
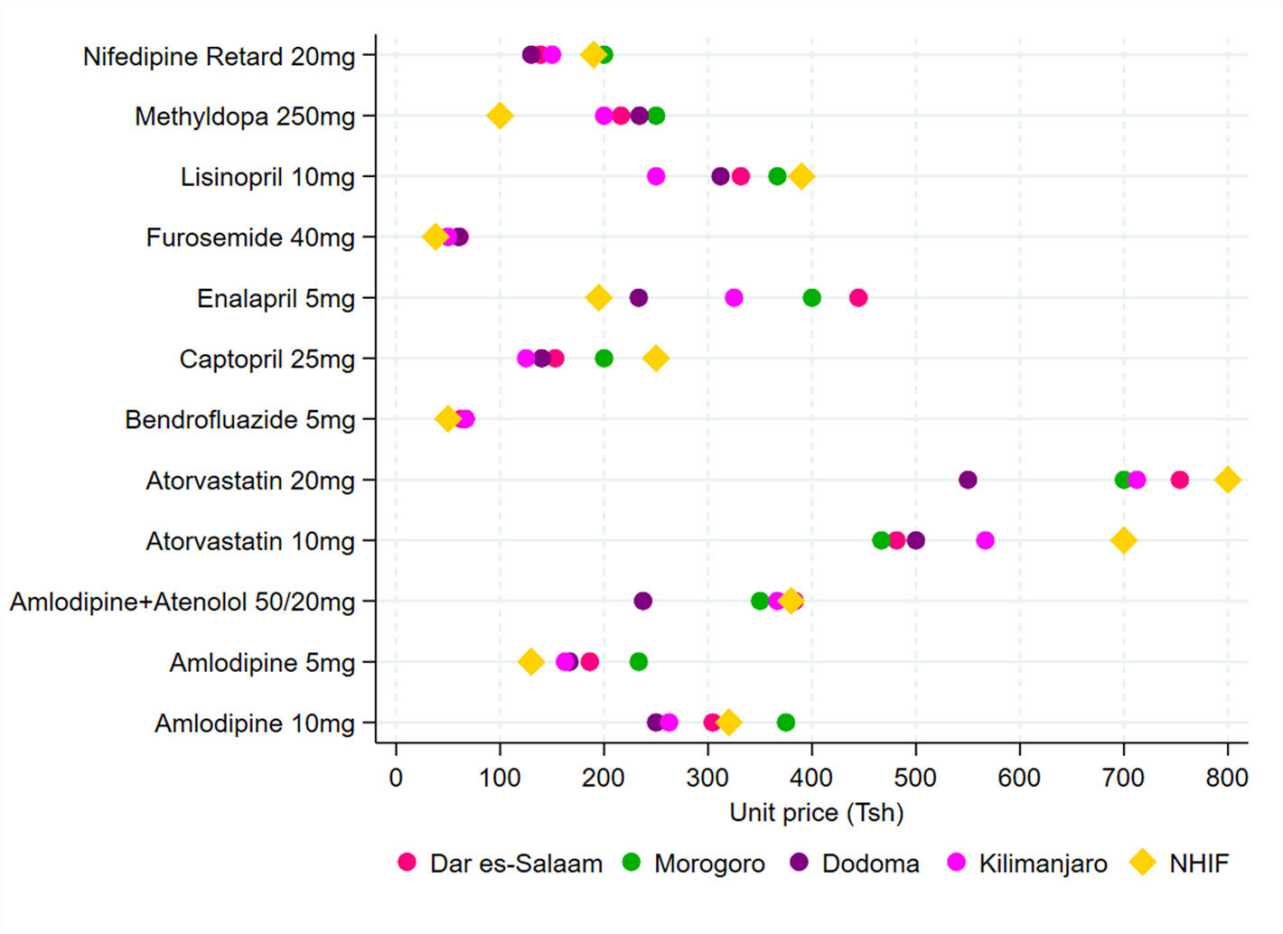
Pharmacy and NHIF prices for medicines used to manage cardiovascular diseases

Figure 3 shows the mean private pharmacy prices for medicines used in the management of cardiovascular diseases across the four regions and NHIF reference prices. We observed more variation in pharmacy prices for relatively more expensive medicines than cheap ones. In between the four study regions, a pair-wise post-hoc Dunn test with Bonferroni adjustment was significant (*p* < 0.05) for amlodipine 10 mg (375.0 Tshs in Morogoro versus 250 Tshs and 262.5 Tsh in Dodoma and Kilimanjaro, respectively); .amlodipine+atenolol 50/20 mg (237.5 Tsh in Dodoma versus 373.3 Tshs in Dar es-Salaam and 366.7 Tsh in Kilimanjaro) and lisinopril 10 mg (366.7 Tshs in Morogoro versus 250 Tsh in Kilimanjaro).

When NHIF prices were compared to pharmacy prices, a pair-wise Dunn test with Bonferroni adjustment was significant (*p* < 0.05) for all the medicines used in the management of cardiovascular diseases except amlodipine 5 mg, atorvastatin 20 mg, and bendrofluazide 5 mg. For nifedipine retard 20 mg, the NHIF price was lower than pharmacy prices in all the regions while for methyldopa 250 mg it was lower than pharmacy prices in all regions except Morogoro. For atorvastatin 10 mg, the NHIF reference price was higher than pharmacy prices in Dar es-Salaam and Morogoro i.e. 700 Tshs versus 481.3 Tsh and 466.7 Tshs, respectively. For captopril 25 mg, the NHIF price (250 Tsh) was higher than pharmacy prices in Dar es-Salaam (153 Tsh), Dodoma (140 Tsh), and Kilimanjaro (125 Tsh) (see Additional file 2).

### Medicines for the management of diabetes

**Fig. 4:**
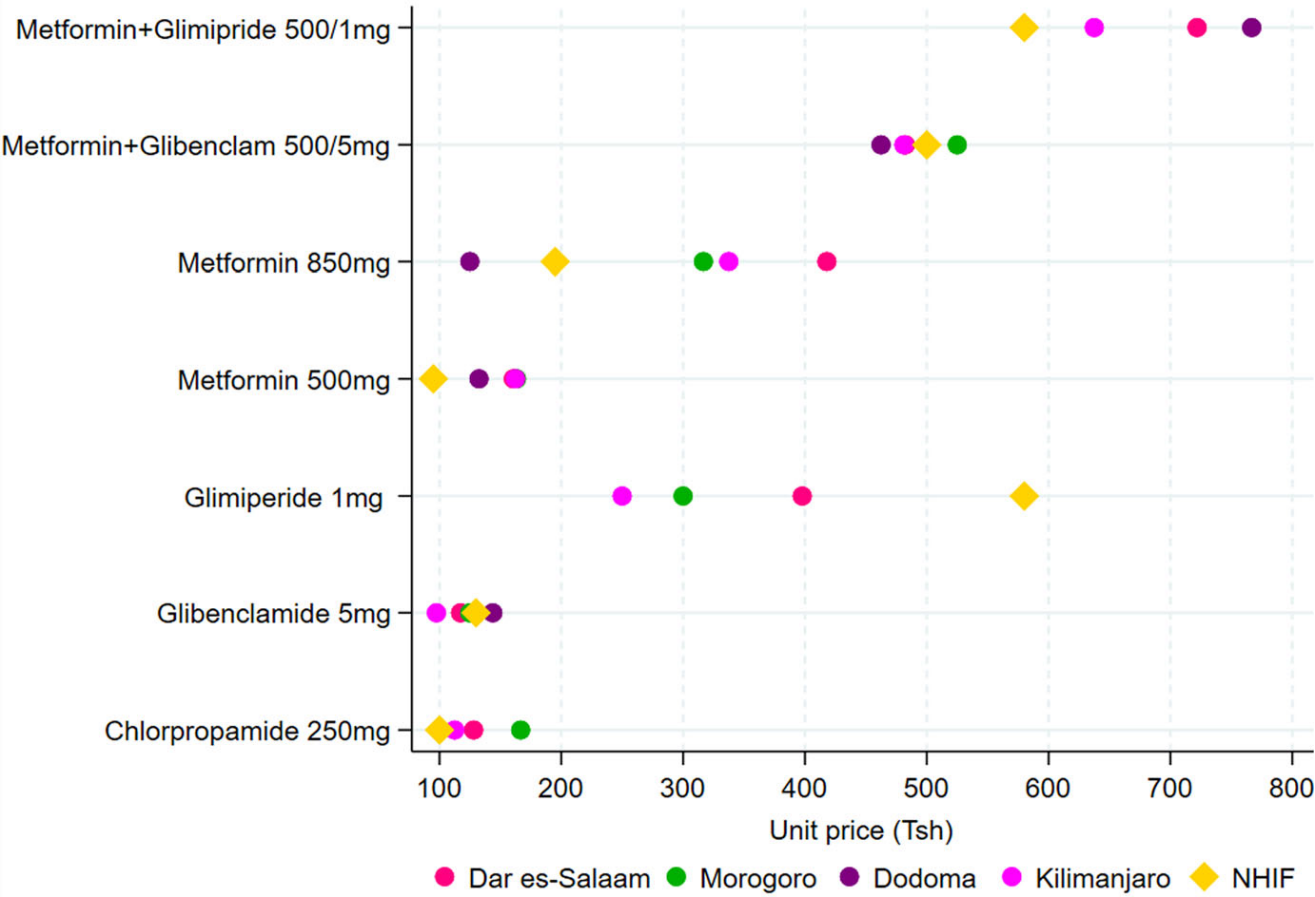
Pharmacy prices and NHIF reference price for antidiabetic medicines

Figure 4 shows the mean pharmacy prices of 7 medicines used for the management of diabetes in the study regions and the NHIF reference prices. There were no significant differences in regional mean pharmacy prices for all antidiabetics medicines surveyed except metformin 850 mg for which pair-wise Dunn test with Bonferroni adjustment indicated a difference between Dar es-Salaam (418 Tsh), Dodoma (125 Tsh), and Kilimanjaro (337.5 Tsh).

Pair-wise comparison to the NHIF reference prices indicated that pharmacy prices for metformin 500 mg were significantly lower in all the regions, while for glimepiride 1 mg the NHIF reference price was significantly higher than the mean pharmacy prices in all regions. NHIF prices were also lower than pharmacy prices for metformin 850 mg tablets in Dar es Salaam and Kilimanjaro regions. Also the NHIF reference price for the combination of metformin 500 mg + glimiperide 1 mg was significantly lower than pharmacy prices in Dar es -Salaam (721.9 Tsh), Dodoma (220 Tsh), and Kilimanjaro (637.5 Tshs) regions. (see Additional file 3).

### Comparison with WHO/HAI-2012 median prices

**Fig. 5:**
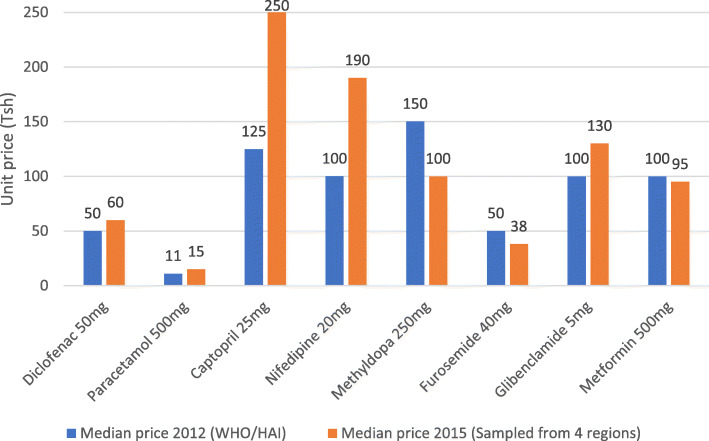
Comparison of median price with WHO/HAI 2012 survey data

Figure 5 shows the comparison of medicines that were surveyed in this study in 2015 and the WHO/HAI nationally surveyed medicines in 2012. The Sign test indicates there was a significant change in price for all medicines except for furosemide 40 mg (*p* < 0.05). The largest increase in the price was observed for captopril 25 mg and nifedipine retard 20 mg and the largest price reduction was observed for methyldopa 250 mg.

## Discussion

The study indicates that there is a large variation in prices of surveyed essential medicines sold in private pharmacies in the sampled four regions in Tanzania. The study has also shown that nearly half of the sampled medicines in the private sector pharmacies are sold at a price that is higher than the reference prices used for reimbursement by the National Health Insurance Fund (NHIF). Anecdotal evidence shows that when patients covered by NHIF visit accredited private pharmacies to fill their prescriptions, they are denied access to the prescribed essential medicines if the reference price is lower than the price in the pharmacy, otherwise they have to co-pay from their pockets the price difference. Therefore, our finding of the large price differences between the pharmacies and NHIF may suggest that insured patients are at risk of being denied access to the prescribed medicines or face high out-of-pocket co-payments.

Retail private sector pharmacies are usually the patient’s first point of contact with the health system in most low- and middle-income countries [26]. They are also the preferred source of medicines for many reasons including frequent stock-out in the public facilities, easy geographical accessibility, shorter waiting times, long opening hours [27, 28]. These premises will continue to be an important source of medicines to patients in these countries in the foreseeable future as the public sector where the poor rely on affordable health care continue to suffer from poor quality of care often characterized by frequent stock-out of essential medicines. Unfortunately, evidence has shown that prices in the private sector are 9–20 times higher than the international reference prices for lowest-priced generics [29]. Therefore, it is important to establish a regulatory mechanism in countries such as Tanzania where such systems are nonexistent to ensure prices of essential medicines are closely monitored and controlled to enhance progress to Universal Health Coverage and Sustainable Development Goals. In the absence of such policies and regulations, the emerging and expanding health insurance schemes will also suffer [30]. Such a rigorous system will enhance access to essential medicines and minimize fraudulent co-payments in the private sector. Evidence from Mali shows that the government was able to correct market imperfection by introducing regulation on medicine pricing, which led to an overall price reduction and improved access to essential medicines in the private pharmacy [31].

In between the 1990s to 2005, about 30% of medicines circulating in the Tanzanian market were produced locally [32]. However, ever since the domestic production of pharmaceuticals has decreased significantly to be replaced with importation. As of the year 2014, pharmaceuticals from domestic production accounted for only about 12% of the market share [33]. More than half of the imported medicines in Tanzania in terms of market value comes from Indian Pharmaceutical companies followed by Egypt. Antibacterial, analgesics, and antimalarial medicines were the top three categories [34]. The inability of local industries to produce enough low-cost generics medicines satisfying Good Manufacturing Practice standards [33], means imports from external sources will continue to dominate the Tanzanian market. It is predicted that by the year 2021 the private sector market value of pharmaceuticals will increase by 28% [34]. A study by Ewen et al. 2016 showed that in a sample of 24 essential medicines, prices in the private sector were 101 and 201% higher than the international reference prices for locally produced and imported medicines, respectively [35]. Lack of effective pricing policies and regulations means prices in the private sector pharmacies will remain higher than international prices, making treatments for chronic conditions such as diabetes and cardiovascular diseases unaffordable to many in low- and middle-income countries [29].

Health Technology Assessment methodology, which provides a systematic way of informing pricing and reimbursement decisions in most high-income countries is relatively a new concept in the developing world [36]. Tanzania also does not have an agency devoted to controlling medicine prices, although it does have one for controlling prices of energy and water utilities [37]. In the absence of such systems, market forces may be the only factor that determines the prices of medicines in the private sector. However, as this study and others have pointed out, market forces alone may not be sufficient to ensure the efficient and equitable distribution of healthcare services, which necessitates government intervention [38].

The NHIF determines the reimbursement rates based on market surveys on the prevailing prices and actuarial evaluations [22]. Apart from this, there is no clear information on how they arrive at the final reimbursement prices of medicines. This underscores the importance of establishing a more transparent and effective pricing system considering that prices in the dominant private market are highly inflated compared to the corresponding international price [29]. Medine prices are also prone to currency fluctuations, and as this study shows vary a lot between regions. Another challenge was that the NHIF reference price list used during the study period was updated more than 3 years ago, which could explain why prices for some medicines were in most cases lower than the prices we found in the private pharmacies. Through the interviews with owners and operators of the pharmacies, we learned that selling medicines at the reference prices would mean incurring losses, which explains why patients were being told to pay the difference or being denied access. Therefore it is important to review the reference price list more frequently through an open and transparent process that engages the key stakeholders.

### Study limitations

This study has several limitations. First, the sample size was relatively small for medicines surveyed in Morogoro, Dodoma, and Kilimanjaro regions. The reason being the small number of accredited pharmacies in these regions. With a small sample size, one can easily reach a wrong conclusion. However, despite this weakness, the findings of this study highlight an important problem that needs further investigation. Second, we did not have enough data to determine factors that are associated with price variation. Hence, we failed to provide answers as to why the prices of some medicines in Dar es-Salaam region were higher than in other regions. We expected lower pharmacy prices in Dar es-Salaam because of market competition and lower transport costs, because it is an import hub and contains more than half of all pharmacies in the country. Third, we were not able to compare the private pharmacy and the NHIF reimbursement prices to the international reference prices, which could have shed more light on how local retail medicine prices compare to the corresponding international prices.

## Conclusion

The study found that medicine prices in private pharmacies vary a lot between the study regions, which raises equity concerns. Also, there was a significant difference between the pharmacy and the NHIF reimbursement prices. Often, when the NHIF reimbursement prices are lower than the private pharmacy prices, patients are asked to pay the difference, which may expose them to fraudulent co-payments that further increases their vulnerability to high out-of-pocket payments or it may delay timely access to the prescribed medicines. Therefore, effective price control policies and regulations for medicines are warranted in Tanzania.

## Supplementary Information

**Additional file 1:** **Table 1.** Mean pharmacy price and NHIF reference prices (in Tsh.) for antipretic medicines.

**Additional file 2:** **Table 2.** Prices (Tsh) of medicines for management of cardiovascular diseases.

**Additional file 3:** **Table 3.** Pharmacy and NHIF prices (Tsh) of medicines used in the management of diabetes.

## Data Availability

The datasets used and/or analyzed in this study are included as additional files, but additional information can be made available by the first author on request.

## Abbreviations

HAI: Health Action International
NCDs: Non-Communicable Diseases
LMIC: Low- and Middle-Income Countries
NHIF: National Health Insurance Fund
WHO: World Health Organization

## References

[1] WHO (2018). Non-communicable diseases-Key facts.

[2] Murray CJ, Global Burden of Disease Study Group (2012). Disability-adjusted life years (DALYs) for 291 diseases and injuries in 21 regions, 1990–2010: a systematic analysis for the global burden of disease study 2010. Lancet.

[3] Geneau R, Stuckler D, Stachenko S, McKee M, Ebrahim S, Basu S, Chockalingham A, Mwatsama M, Jamal R, Alwan A (2010). Raising the priority of preventing chronic diseases: a political process. Lancet.

[4] Amuna P, Zotor FB (2008). Epidemiological and nutrition transition in developing countries: impact on human health and development. Proc Nutr Soc.

[5] Reddy KS, Yusuf S (1998). Emerging epidemic of cardiovascular disease in developing countries. Circulation.

[6] Global, regional, and national comparative risk assessment of 79 behavioural, environmental and occupational, and metabolic risks or clusters of risks, 1990–2015: a systematic analysis for the global burden of disease study 2015. Lancet. 2016;388(10053):1659–724. 10.1016/S0140-6736(16)31679-8 PMC5388856 27733284

[7] Beaglehole R, Bonita R, Horton R, Adams C, Alleyne G, Asaria P, Baugh V, Bekedam H, Billo N, Casswell S (2011). Priority actions for the non-communicable disease crisis. Lancet.

[8] Wolff JL, Starfield B, Anderson G (2002). Prevalence, expenditures, and complications of multiple chronic conditions in the elderly. Arch Intern Med.

[9] Wagstaff A, Flores G, Hsu J, Smitz MF, Chepynoga K, Buisman LR, van Wilgenburg K, Eozenou P (2018). Progress on catastrophic health spending in 133 countries: a retrospective observational study. Lancet Glob Health.

[10] Spaan E, Mathijssen J, Tromp N, McBain F, ten Have A, Baltussen R (2012). The impact of health insurance in Africa and Asia: a systematic review. Bull World Health Organ.

[11] Fadlallah R, El-Jardali F, Hemadi N, Morsi RZ, Abou Samra CA, Ahmad A, Arif K, Hishi L, Honein-AbouHaidar G, Akl EA (2018). Barriers and facilitators to implementation, uptake and sustainability of community-based health insurance schemes in low- and middle-income countries: a systematic review. Int J Equity Health.

[12] Dror DM, Hossain SA, Majumdar A, Pérez Koehlmoos TL, John D, Panda PK (2016). What factors affect voluntary uptake of community-based health insurance schemes in low- and middle-income countries? A systematic review and meta-analysis. PLoS One.

[13] United Nations (2013). Social Protection and Universal Health Coverage (A/RES/67/81).

[14] WHO (2011). The world medicine situation: medicines prices, availability and affordability.

[15] WHO (2015). WHO guideline on country pharmaceutical pricing policies.

[16] Kaplan WA, Ritz LS, Vitello M, Wirtz VJ (2012). Policies to promote use of generic medicines in low and middle income countries: a review of published literature, 2000-2010. Health Policy.

[17] Kanavos P, Fontrier AM, Gill J, Efthymiadou O (2020). Does external reference pricing deliver what it promises? Evidence on its impact at national level. Eur J Health Econ.

[18] Tantivess S, Chalkidou K, Tritasavit N, Teerawattananon Y (2017). Health Technology Assessment capacity development in low- and middle-income countries: Experiences from the international units of HITAP and NICE. F1000Res.

[19] United Republic of Tanzania. 2012 Population housing census, vol. 2013. Dar es Salaam.

[20] Rutta E, Senauer K, Johnson K, Adeya G, Mbwasi R, Liana J, Kimatta S, Sigonda M, Alphonce E (2009). Creating a new class of pharmaceutical services provider for underserved areas: the Tanzania accredited drug dispensing outlet experience. Prog Community Health Partnersh.

[21] The National Health Insurance Fund-Profile https://www.nhif.or.tz/pages/profile#gsc.tab=0. Accessed 6 July 2020).

[22] Lee B, Tarimo K, Dutta A. Analysis of cost escalation at the National Health Insurance Fund in Tanzania-policy brief. Health Policy Plus. 2018.

[23] World Health Organization/Health Action International. Measuring medicine prices, availability, affordability and price components. 2nd ed. Geneva; 2008.

[24] Ministry of Health and Social Welfare. Standard Treatment Guidelines and National Essential Medicine List-Tanzania Mainland. 4th ed: Dar es Salaam; 2013.

[25] Dinno A (2015). Non-parametric pairwise multiple comparisons in independent groups using Dunn’s test. Stata J.

[26] Smith F (2009). Private local pharmacies in low- and middle-income countries: a review of interventions to enhance their role in public health. Tropical Med Int Health.

[27] Babar ZU, Ramzan S, El-Dahiyat F, Tachmazidis I, Adebisi A, Hasan SS (2019). The availability, pricing, and affordability of essential diabetes medicines in 17 low-, middle-, and high-income countries. Front Pharmacol.

[28] Embrey M, Vialle-Valentin C, Dillip A, Kihiyo B, Mbwasi R, Semali IA, Chalker JC, Liana J, Lieber R, Johnson K (2016). Understanding the role of accredited drug dispensing outlets in Tanzania's health system. PLoS One.

[29] Cameron A, Ewen M, Ross-Degnan D, Ball D, Laing R (2009). Medicine prices, availability, and affordability in 36 developing and middle-income countries: a secondary analysis. Lancet.

[30] Carapinha JL, Ross-Degnan D, Desta AT, Wagner AK (2011). Health insurance systems in five sub-Saharan African countries: medicine benefits and data for decision making. Health Policy.

[31] Maïga D, Williams-Jones B (2010). Assessment of the impact of market regulation in Mali on the price of essential medicines provided through the private sector. Health Policy.

[32] MoHSW (2006). The National Medicine Policy.

[33] EAC (2014). The 2nd East African Community (EAC) Regional Pharmaceutical Manufacturing Plan of Action (RPMPOA) 2017–2027.

[34] Wande DP, Sangeda RZ, Tibalinda P, Mutta IK, Mkumbwa S, Bitegeko A, Kaale E (2019). Pharmaceuticals imports in Tanzania: overview of private sector market size, share, growth and projected trends to 2021. PLoS One.

[35] Ewen M, Kaplan W, Justin-Temu M (2016). Prices and availability of locally produced and imported medicines in Tanzania.

[36] WHO (2015). 2015 Global survey on health technology assessment by National Authorities.

[37] The Government of the United Republic of Tanzania. The Energy and Water Utilities Regulatory Authority Act. Dar es Salaam; 2006.

[38] Nichols LM, Ginsburg PB, Berenson RA, Christianson J, Hurley RE (2004). Are market forces strong enough to deliver efficient health care systems? Confidence is waning. Health Aff (Project Hope).

