# Supplementary material for: Prices of medicines for the management of pain, diabetes and cardiovascular diseases in private pharmacies and the national health insurance in Tanzania
Source: Int J Equity Health. 2020 Nov 10;19:203. doi: 10.1186/s12939-020-01319-9 (PMC7653889; doi:10.1186/s12939-020-01319-9)
Supplement: Supplementary file 1 — Additional file 1: Table 1. Mean pharmacy price and NHIF reference prices (in Tsh.) for antipretic medicines. [file 12939_2020_1319_MOESM1_ESM.docx]

Table 1: Mean pharmacy price and NHIF reference prices (in Tsh.) for antipretic medicines

|  | Diclofenac 50 mg | Ibuprofen 200mg | Indomethacin 25mg | Paracetamol 500mg | Diclofenac 100mg | Ketoprofen 50mg | Ketotifen 1mg | Mefenamic acid 250 mg | Tramadol 50mg |
| --- | --- | --- | --- | --- | --- | --- | --- | --- | --- |
| Dar es Salaam | 55.0^a^ | 53.9^a^ | 53.2^a^ | 30.4^a^ | 157.1^a^ | 337.5 ^a^ | 250.0 | 267.2 ^a^ | 213.2^a^ |
| Morogoro | 56.3^b^ | 42.8^b^ | 44.0^b^ | 26.3^b^ | 250.0^b^ | 400.0 ^b^ | 250.0 | 125.0 ^b^ | 268.0^b^ |
| Dodoma | 62.5^c^ | 66.7^c^ | 43.3 | 43.8^c^ | 175.4^c^ | 400.0 ^c^ | 160.0 ^c^ | 100.0 ^c^ | 166.7^c^ |
| Kilimanjaro | 56.3^d^ | 50.0^d^ | 42.5 | 22.5 | 150.0^d^ | 250.0 | 212.5 | 212.5 ^d^ | 218.8^d^ |
| NHIF price | 60.0^e^ | 20.0^e^ | 25.0^e^ | 15.0^d^ | 200.0^e^ | 200.0 ^e^ | 260.0^e^ | 195.0 ^e^ | 390.0^e^ |
| **Kruskal-Wallis test** | χ²=15.57, p=0.003 | χ²=31.54, p=0.0001 | χ²=19.28, p=0.0007 | χ²=25.52, p=0.0001 | χ²=24.68, p=0.0001 | χ²=25.00, p=0.0001 | χ²=10.49, p=0.03 | χ²=18.49, p=0.01 | χ²=44.74, p=0.0001 |
| **Pair-wise Dunn test**  p<0.05 | e>a (p=000) | e<a (p=0.000)  e<b (p=0.031)  e<c (p=0.002)  e<d (p=0.007) | e<a (p=0.000)  e<b (p=0.04) | e<a (p=0.000)  e<b (p=0.047)  e<c (p=0.000) | a<b (p=0.01)  b>d (p=0.008)  e>a (p=0.000)  e>d (p=0.001) | e<a (p=0.000)  e<b (p=0.01)  e<c (p=0.01) | e>c (p=0.04) | a>b (p=0.01)  a>c (p=0.001)  d>c (p=0.01)  e>c (p=0.017) | e>a (p=0.000)  e>c (p=0.001)  e>d (p=0.005) |
