# Supplementary material for: Prices of medicines for the management of pain, diabetes and cardiovascular diseases in private pharmacies and the national health insurance in Tanzania
Source: Int J Equity Health. 2020 Nov 10;19:203. doi: 10.1186/s12939-020-01319-9 (PMC7653889; doi:10.1186/s12939-020-01319-9)
Supplement: Supplementary file 2 — Additional file 2: Table 2. Prices (Tsh) of medicines for management of cardiovascular diseases. [file 12939_2020_1319_MOESM2_ESM.docx]

Table 2: Prices (Tsh) of medicines for management of cardiovascular diseases

|  | **Name of Medicine** | **Dar es Salaam**  **region** | **Dodoma**  **region** | **Morogoro**  **region** | **Kilimanjaro**  **region** | **NHIF Price** | **Kruskal-Wallis test** | **Pairs-wise Dunn test** (p<0.05) |
| --- | --- | --- | --- | --- | --- | --- | --- | --- |
| 1 | Amlodipine 5mg | 186.3 | 166.7 | 233.3 | 162.5 | 130.0 | χ²=7.870,  p=0.096 | - |
| 2 | Amlodipine 10mg | 304.4^a^ | 250.0^b^ | 375.0^c^ | 262.5^d^ | 320.0^e^ | χ²=28.770  p=0.0001 | b<c (p=0.007)  d<c (p=0.013)  e>a (p=0.000)  e>b (p=0.000)  e>d (p=0.003) |
| 3 | Amlodipine + Atenolol 50/20mg | 383.3^a^ | 237.5^b^ | 350.0 | 366.7^d^ | 380.0^e^ | χ²=13.113  p=0.0107 | b<a (p=0.033)  b<d (p=0.009)  e>b (p=0.000) |
| 4 | Captopril 25mg | 153.0^a^ | 140.0^b^ | 200^c^ | 125.0^d^ | 250.0^e^ | χ²=29.790  p=0.0001 | e>a (p=0.000)  e>b (p=0.002)  e>d (p=0.003) |
| 5 | Enalapril 5mg | 444.7^a^ | 233.3^b^ | 400.0^c^ | 325.0^d^ | 195.0^e^ | χ²=39.932  p=0.0001 | e<a (p=0.000)  e<c (p=0.006)  e<d (p=0.012) |
| 6 | Lisinopril 10mg | 331.5 | 312.5 | 366.7.0^c^ | 250.0^d^ | 390.0^e^ | χ²=14.315  p=0.0064 | c>d (p=0.011)  e>d (p=0.001) |
| 7 | Methyldopa 250mg | 216.3^a^ | 234.0^b^ | 250.0* | 200.0^d^ | 100.0^e^ | χ²=40.557  p=0.0001 | e<a (p=0.000)  e<b (p=0.000)  e<d (p=0.038) |
| 8 | Nifedipine Retard 20mg | 138.9^a^ | 130.0^b^ | 200.0 | 150.0^d^ | 190.0^e^ | χ²=23.764  p=0.0001 | e>a (p=0.000)  e>b (p=0.027)  e>d (p=0.033) |
| 9 | Atorvastatin 10mg | 481.3^a^ | 500.0 | 466.7^c^ | 566.7 | 700.0^e^ | χ²=21.478  p=0.0001 | e>a (p=0.000)  e>c (p=0.020) |
| 10 | Atorvastatin 20mg | 753.8 | 550.0** | 700.0 | 712.5.0^d^ | 800.0^e^ | χ²=9.551  p=0.05 | - |
| 11 | Bendrofluazide 5mg | 62.5 | 66.7.0 | 50.0 | 66.7 | 50.0 | χ²=2.835  p=0.5858 | - |
| 12 | Furosemide 40mg Tab | 61.0^a^ | 60.0^b^ | 50.0^c^ | 50.0^d^ | 38.0^e^ | χ²=42.031  p=0.001 | e<a (p=0.000)  e<b (p=0.016)  e<c (p=0.000)  e<d (p=0.016) |

* small sample size made the dunn test insignificant

** Standard deviation was too high
