# Supplementary material for: Prices of medicines for the management of pain, diabetes and cardiovascular diseases in private pharmacies and the national health insurance in Tanzania
Source: Int J Equity Health. 2020 Nov 10;19:203. doi: 10.1186/s12939-020-01319-9 (PMC7653889; doi:10.1186/s12939-020-01319-9)
Supplement: Supplementary file 3 — Additional file 3: Table 3. Pharmacy and NHIF prices (Tsh) of medicines used in the management of diabetes. [file 12939_2020_1319_MOESM3_ESM.docx]

Table 3: Pharmacy and NHIF prices (Tsh) of medicines used in the management of diabetes

|  | **Antidiabetic medicines** | **Dar es Salaam**  **region** | **Morogoro**  **region** | **Dodoma**  **region** | **Kilimanjaro**  **region** | **NHIF price** | **Kruskal-Wallis test** | **Pair-wise Dunn test**  p<0.05 |
| --- | --- | --- | --- | --- | --- | --- | --- | --- |
| 1 | Chlorpropamide 250mg Tab | 128.1 | 166.7 | 100.0 | 112.5 | 100.0 | χ²=5.068, p=0.280 | - |
| 2 | Glibenclamide 5mg Tab | 117.3 | 125.0 | 143.8 | 97.5 | 130.0 | χ²=5.542, p=0.236 | - |
| 3 | Glimiperide 1mg Tab | 397.8^a^ | 300.0^b^ | 80.0* | 250.0^d^ | 580.0^e^ | χ²=22.405, p=0.0002 | e>a (p=0.000)  e>b (p=0.024)  e>d (p=0.01) |
| 4 | Metformin 500mg Tab | 160.7^a^ | 163.3^b^ | 132.5^c^ | 162.5^d^ | 95.0^e^ | χ²= 27.00, p=0.0001 | e>a (p=0.000)  e>b (p=0.013)  e>c (p=0.023)  e>d (p=0.006) |
| 5 | Metformin 850mg Tabs | 418.0^a^ | 316.7^b^ | 125.0^c^ | 337.5^d^ | 195.0^e^ | χ²=37.351, p=0.0001 | c<a (p=0.000)  c<d (p=0.004)  e<a (p=0.000)  e<d (p=0.009) |
| 6 | Metformin +Glibenclamide 500/5mg Tab | 482.5 | 525.0 | 462.5 | 481.3 | 500.0 | χ²=4.679, p=0.3218 | - |
| 7 | Metformin+Glimipride 500/1mg Tab | 721.9^a^ | 800.0^*^ | 766.7^c^ | 637.5^d^ | 580.0^e^ | χ²=42.20, p=0.0001 | e>a (p=0.000)  e>c (p=0.002)  e>d (p= 0.017) |

* Only one observation available
